# A Low-Polymer High-Temperature Water-Based Fracturing Gel Enabled by a Dual-Ligand Organic Zirconium Crosslinker: An Experimental Study

**DOI:** 10.3390/gels12090829

**Published:** 2026-09-10

**Authors:** Fei Liu, Xuewu Wang, Xiaqing Li, Peng Tao, Boyang Shen, Yuyu Zhang, Shaocan Dong, Yongfei Li

**Affiliations:** 1 College of Petroleum Engineering, Shandong Institute of Petroleum and Chemical Technology, Dongying 257061, China; 2 Efficient Exploration and Development of Oil and Gas Reservoirs and the Integration of Geology and Engineering Shandong Provincial Engineering Research Center, Dongying 257061, China; 3 Shandong Key Laboratory of Shale Oil Exploration and Development in Continental Faulted Basin, Dongying 257015, China; 4 Shandong Key Laboratory of Green Electricity & Hydrogen Science and Technology, Dongying 257061, China; 5 Petroleum Engineering Technology Research Institute of Shengli Oilfield Company, SINOPEC, Dongying 257000, China; 6 Shaanxi University Engineering Research Center of Oil and Gas Field Chemistry, Xi’an Petroleum University, Xi’an 710065, China; 7 College of Pipeline and Civil Engineering, China University of Petroleum (East China), Qingdao 266580, China

**Keywords:** organic zirconium, coordination crosslinking, high-temperature rheology, low-polymer fracturing fluid, proppant suspension

## Abstract

This experimental study aimed to develop and evaluate a low-polymer, high-temperature water-based fracturing gel using a dual-ligand organic zirconium crosslinker regulated by sodium lactate and ethylene glycol. The crosslinker was selected by ligand screening and single-factor optimization using the apparent viscosity of LX641 gels as the primary response, and was characterized by FTIR and electron microscopy. Gelation, salt response, high-temperature shear rheology, oscillatory and steady-shear behavior, static fluid loss, gel breaking, proppant suspension, and core-permeability damage were then evaluated. The selected zirconium oxychloride octahydrate/sodium lactate/ethylene glycol/water/NaOH mass ratio was 10:6:4:15:1.2, with synthesis at 55 °C, pH 7, for 4 h. A 0.2 wt.% LX641 gel at a base-fluid/crosslinker-solution volume ratio of 100:0.5 retained an apparent viscosity of 246.84 mPa·s at 100 min after heating to 190 °C within 25 min and shearing at 190 °C for the remaining 75 min at 170 s^−1^. The system also showed no visible settling of 10 wt.% 30-mesh ceramic proppant after 12 h, core-permeability damage of 11.98–13.65%, and visually clear broken fluid within 2 h using 0.01 wt.% ammonium persulfate at 70 °C. The results indicate that sodium-lactate/ethylene-glycol regulation can support substantial high-temperature viscosity retention at only 0.2 wt.% polymer loading, providing a low-polymer alternative for further development of high-temperature zirconium-crosslinked fracturing gels.

## 1. Introduction

Hydraulic fracturing fluids transfer pressure, generate and extend fractures, and transport proppant into the created fracture network. Their viscosity, elasticity, thermal stability, and fluid-loss behavior therefore influence both fracture development and proppant placement [1,2,3]. As development moves toward deep and ultra-deep reservoirs, fluid systems may experience high temperature, high shear, and saline formation water. These conditions accelerate polymer-chain degradation, reduce hydrodynamic volume, disrupt physical entanglements, and complicate control of the crosslinking process [1,2].

Guar and guar derivatives remain important thickeners because of their strong gelation and proppant-carrying ability. However, natural-polymer systems can generate insoluble residues and can require relatively high polymer loading. Synthetic acrylamide-based, hydrophobically associating, and polymer–nanocomposite systems provide alternatives because their backbones and functional comonomers can be designed for thermal and electrolyte tolerance [3,4,5,6,7,8]. In particular, AMPS-containing polymers retain hydration under saline conditions, acrylic acid provides carboxylate coordination sites, and cationic comonomers can modify chain conformation and solution compatibility. Nevertheless, the performance of such polymers at low concentration depends strongly on the chemistry and release kinetics of the crosslinker.

Organic zirconium complexes are attractive high-temperature crosslinkers because zirconium species can coordinate with oxygen-containing groups on polymer chains and form networks that are more thermally persistent than conventional borate-crosslinked gels [9,10,11,12,13,14]. The ligand environment controls zirconium hydrolysis, ligand exchange, gelation time, and the balance between insufficient and excessive crosslinking. Nanoparticle and colloidal crosslinker studies further show that crosslinker architecture and network morphology can influence thermal stability, filtration control, and rheological strength [15,16,17,18]. Recent delayed, weighted, and double-network gel systems have emphasized the combined importance of controlled gelation, low polymer loading, high-temperature viscosity retention, and reduced formation damage [19,20,21,22].

This work develops an organic zirconium crosslinker using sodium lactate and ethylene glycol as complementary ligands. The crosslinker formulation was selected through broad ligand screening and single-factor process optimization, and was subsequently evaluated with LX641, an AM/AMPS/DMDAAC/AA tetrapolymer. Gelation kinetics, programmed temperature–shear response, oscillatory and steady-shear rheology, salt response, static fluid loss, gel breaking, proppant suspension, and core-permeability damage were systematically examined. The central objective was to determine whether controlled zirconium coordination could support a useful gel network at polymer concentrations as low as 0.2–0.3 wt.% under severe thermal and shear conditions.

The specific contribution of the present study is the use of sodium lactate and ethylene glycol as complementary ligands to regulate an organic zirconium crosslinker for an AM/AMPS/DMDAAC/AA polymer at unusually low polymer loading. For context, an organic-zirconium-crosslinked HPAM system reported by Xin et al. used 0.6 wt.% HPAM and retained 61.43 mPa·s at 200 °C and 100 s^−1^ [9], whereas a supramolecular double-network system reported by Huang et al. used 0.4 wt.% polymer and 0.1 wt.% Zr crosslinker and retained 66 mPa·s after 2 h at 200 °C and 170 s^−1^ [22]. The present 0.2 wt.% LX641 formulation retained 246.84 mPa·s at the end of a 100 min programmed test reaching 190 °C at 170 s^−1^. Because the test protocols and polymer chemistries differ, these values should not be interpreted as a direct ranking of performance; rather, they place the low-polymer design investigated here within the current range of high-temperature zirconium-crosslinked gel technologies.

## 2. Results and Discussion

### 2.1. Ligand Screening and Crosslinker Optimization

Preliminary screening compared single-, dual-, and ternary-ligand zirconium formulations. The sodium lactate/ethylene glycol formulation produced a clear crosslinker solution and a coherent hanging gel with LX641. As summarized in Table 1, at the best crosslinker dosage evaluated during this screening, its gel viscosity (460.8 mPa·s) exceeded those obtained with sodium lactate alone, sodium lactate/glycerol, ethylene glycol/glycerol, and the sodium lactate/ethylene glycol/glycerol formulation. The comparison supports selection of the dual-ligand formulation within the screened set. Because the initial screen used formulation-specific synthesis conditions and qualitative gel observations, it should be interpreted as a practical screening result rather than definitive molecular proof of ligand synergy.

After ligand selection, the sodium lactate amount, ethylene glycol amount, reaction time, synthesis temperature, and synthesis pH were varied individually. The tested ranges and selected conditions are summarized in Table 2. The apparent viscosity of the resulting LX641 gels was used as the primary response variable for single-factor optimization. This sequential approach was selected to establish practical synthesis windows after broad ligand screening; interactions among variables were not quantified and are considered in the limitations of this study.

**Figure 1:**
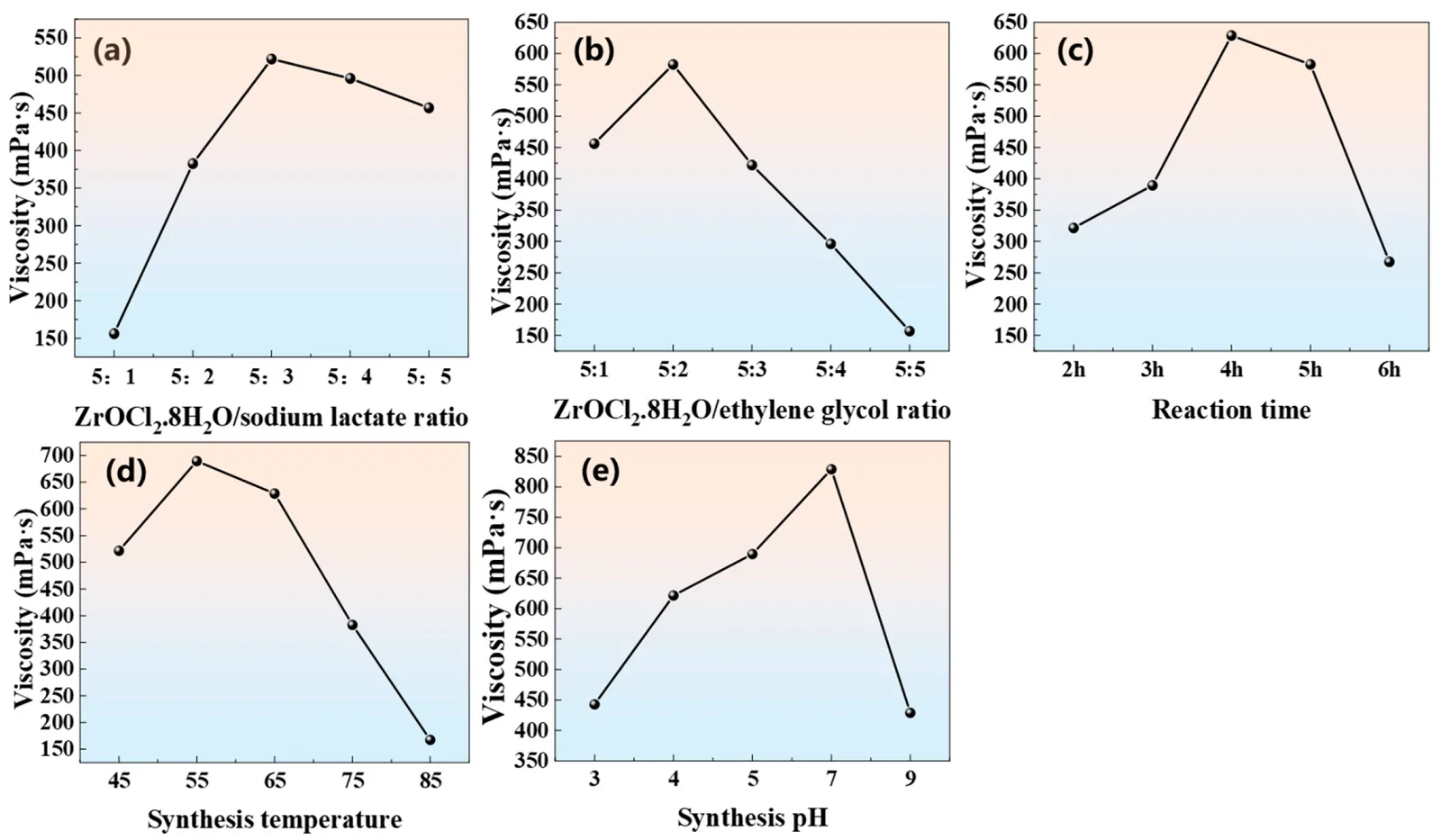
Effects of synthesis variables on the apparent viscosity of LX641 gels: (**a**) zirconium oxychloride/sodium lactate ratio; (**b**) zirconium oxychloride/ethylene glycol ratio; (**c**) reaction time; (**d**) temperature; and (**e**) pH.

As illustrated in Figure 1, a zirconium oxychloride/sodium lactate mass ratio of 5:3 and a zirconium oxychloride/ethylene glycol mass ratio of 5:2 produced the highest apparent viscosity within their respective series. The viscosity response also passed through maxima at 4 h, 55 °C, and pH 7. Longer reaction times, higher synthesis temperatures, or strongly alkaline synthesis conditions reduced gel viscosity, consistent with changes in ligand exchange, hydrolysis, and the availability of reactive zirconium species [10,11,12,13,14]. The selected overall raw-material mass ratio was zirconium oxychloride octahydrate/sodium lactate/ethylene glycol/water/NaOH = 10:6:4:15:1.2. Because the optimization was performed by changing one factor at a time, these selected levels represent practical local optima rather than a statistically resolved global optimum. Independent replicate measurements were not available for the single-factor optimization datasets; therefore, statistical variability is not reported for these data.

### 2.2. Spectroscopic and Morphological Characterization

**Figure 2:**
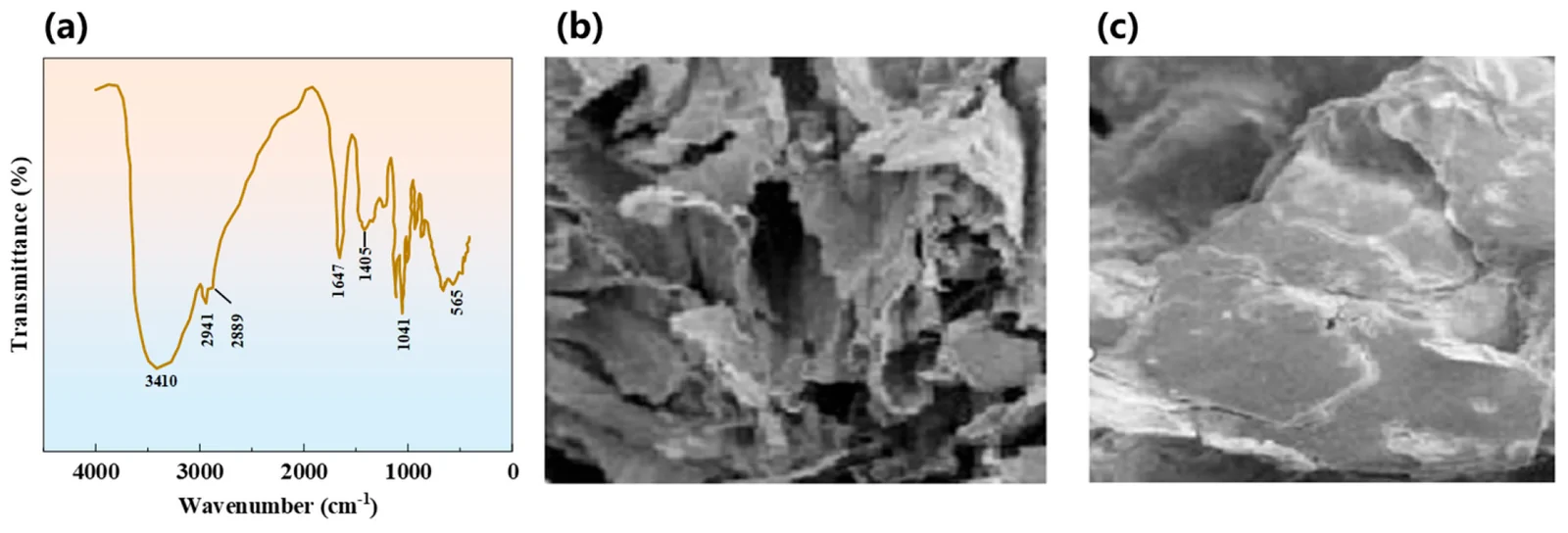
FTIR spectrum (**a**) and SEM images of the vacuum-dried organic zirconium crosslinker at 200× (**b**) and 1000× (**c**).

As shown in Figure 2a, the FTIR spectrum of the dried crosslinker contained a broad O–H stretching band near 3410 cm^−1^, C–H stretching bands at 2941 and 2889 cm^−1^, carboxylate bands at 1647 and 1405 cm^−1^, and a C–O band near 1041 cm^−1^. A low-wavenumber band near 565 cm^1^ was assigned to a Zr–O vibration. Organic-zirconium crosslinker studies have likewise associated changes in carboxylate bands together with low-wavenumber Zr–O features with coordination between zirconium and oxygen-donor ligands [10,11,12,13,14]. The present FTIR spectrum is therefore consistent with zirconium–carboxylate/polyol coordination, but the comparison is supportive rather than structure-determining because band positions can vary with ligand composition, hydration, and sample preparation. FTIR alone does not establish a unique zirconium nuclearity, ligand-exchange pathway, or coordination geometry.

As shown in Figure 2b,c, SEM images of the vacuum-dried crosslinker revealed dense aggregates containing distributed pores. The images characterize the morphology of the dried material but do not directly resolve the structure of the crosslinker in aqueous solution. Accordingly, the pore morphology was not used to infer solution-phase specific surface area or mass-transfer behavior. Molecular-level assignment of zirconium nuclearity and coordination geometry would require complementary solution-state or surface-sensitive characterization.

### 2.3. Gel Formulation and Temperature-Dependent Gelation

The LX641 base fluid was adjusted to pH 8 before addition of the organic zirconium crosslinker. Throughout this article, a ratio written as 100:x denotes the volume ratio of base fluid to crosslinker solution. Increasing the ratio from 100:0.2 to 100:0.8 shortened gelation from 21.6 to 1.5 min. A volume ratio of 100:0.5 was selected for the general formulation because it produced a coherent gel within 2.8 min without using the highest crosslinker dosage. A base-fluid concentration of 0.3 wt.% was selected for the engineering-property tests as a compromise between low polymer loading and stable gel formation, while a 0.2 wt.% base fluid was examined in targeted high-temperature tests.

Gelation was thermally activated over 30–80 °C. As shown in Table 3, the gelation time decreased from 2.9 min at 30 °C to 0.8 min at 80 °C. The acceleration is consistent with faster ligand exchange and more frequent contact between reactive zirconium species and polymer coordination sites. The data demonstrate temperature-sensitive gelation but do not, by themselves, establish reversible bond exchange or self-healing.

### 2.4. Salt Response and High-Temperature Shear Stability

**Figure 3:**
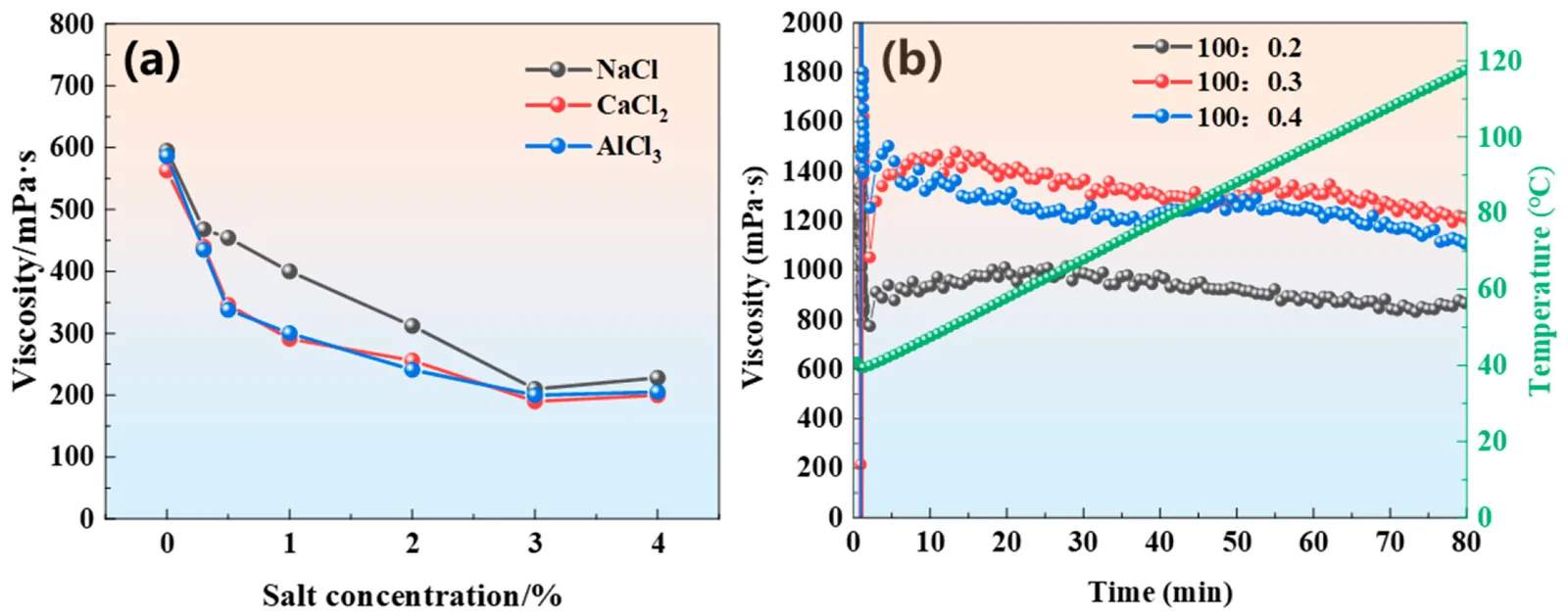
Salt response and crosslinker-dosage effects: (**a**) apparent viscosity of 0.3 wt.% LX641 gels in 0–4 wt.% salt solutions; (**b**) temperature-shear profiles of 0.6 wt.% LX641 gels at volume ratios of 100:0.2, 100:0.3, and 100:0.4. Shear rate: 170 s^−1^.

As shown in Figure 3a, addition of NaCl, CaCl_2_, or ALCl_3_ reduced the apparent viscosity of the crosslinked gel, with the strongest reduction observed for the trivalent cation and the weakest for Na^+^. The order Al^3+^ > Ca^2+^ > Na^+^ is consistent with stronger electrostatic screening and more pronounced ion-mediated changes in polymer conformation as cation valence increases [4,5,6,7,9]. Across the tested concentration range of 0–4 wt.%, the gels retained substantial apparent viscosity. Because a defined failure criterion was not reached within this range, the results are reported as salt response within the tested interval rather than as an absolute salt-tolerance limit.

Uncrosslinked LX641 solutions exhibited a continuous decrease in apparent viscosity during simultaneous heating and shearing because elevated temperature and mechanical shear weakened physical chain entanglements and reduced the hydrodynamic volume of the polymer molecules. In the uncrosslinked base-fluid tests, the temperature was increased from 30 to 90 °C over 60 min under a constant shear rate of 170 s^−1^. Chemical crosslinking substantially altered this response. For gels prepared with 0.6 wt.% LX641, base-fluid/crosslinker-solution volume ratios of 100:0.2, 100:0.3, and 100:0.4 were evaluated while the temperature was increased from 100 to 120 °C and subsequently maintained at 120 °C under 170 s^−1^. As shown in Figure 3b–d, the formulation with a volume ratio of 100:0.3 exhibited the highest terminal apparent viscosity, reaching approximately 1269 mPa·s. The increase in viscosity during heating is attributed to thermally accelerated zirconium–polymer coordination and continued development of the crosslinked network rather than to intrinsic thermal thickening of the uncrosslinked LX641 solution [4,5,6,7,8,19,20,21,22].

**Figure 4:**
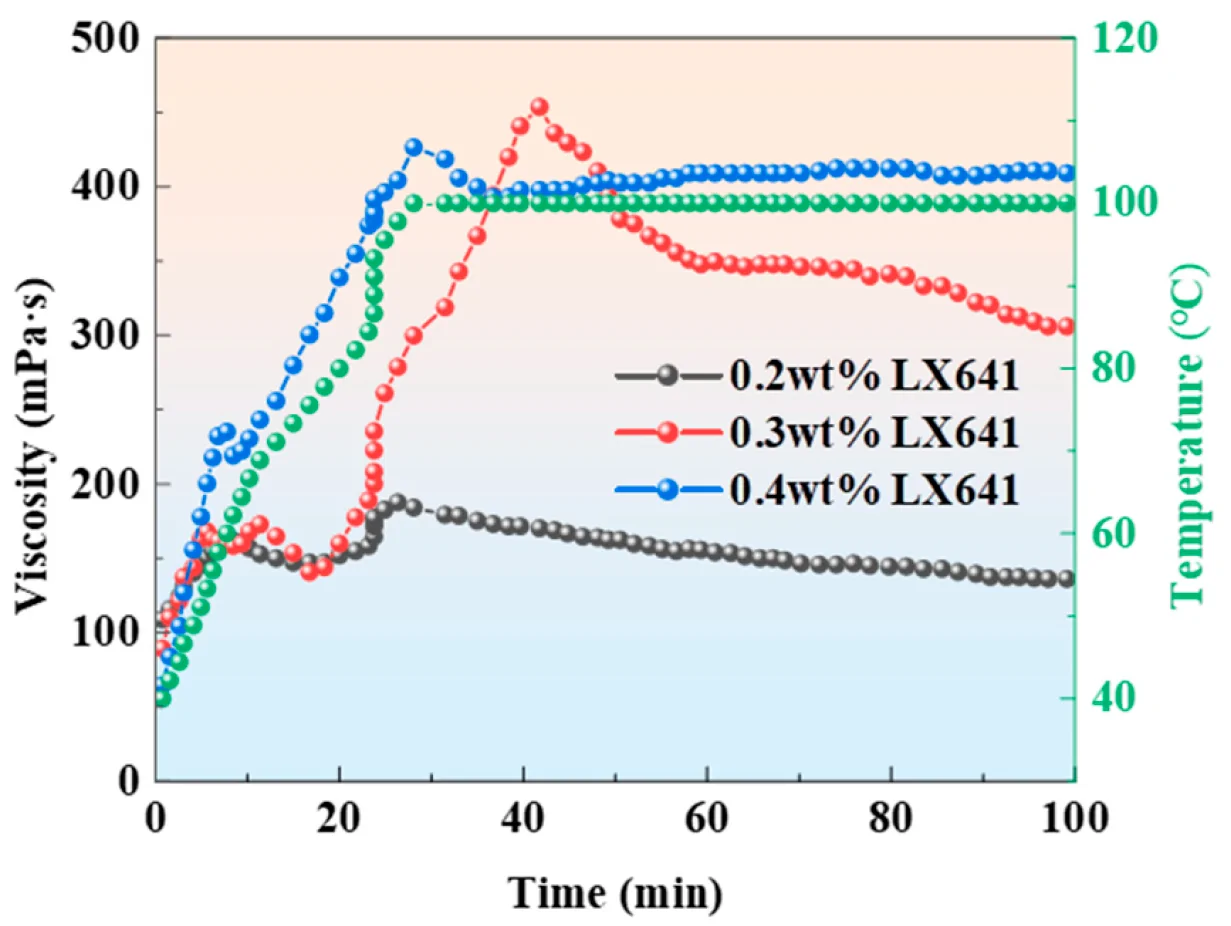
Temperature-shear profiles of 0.2, 0.3, and 0.4 wt.% LX641 gels at a volume ratio of 100:0.3. Test conditions: 30–120 °C, 100 min, and 170 s^−1^.

The effect of polymer concentration was further examined using LX641 concentrations of 0.2, 0.3, and 0.4 wt.% at a fixed base-fluid/crosslinker-solution volume ratio of 100:0.3. During these 100 min programmed tests, the temperature was increased from 30 to 120 °C under a constant shear rate of 170 s^−1^. As shown in Figure 4, the apparent viscosities recorded at the end of the 100 min tests were approximately 130, 280, and 320 mPa·s for the 0.2, 0.3, and 0.4 wt.% LX641 gels, respectively. Although the apparent viscosity decreased as the LX641 concentration was reduced, the gel containing only 0.2 wt.% polymer retained approximately 130 mPa·s at 100 min under the stated temperature-shear program. These results indicate that the organic zirconium crosslinker can support a measurable crosslinked network at relatively low polymer loading.

**Figure 5:**
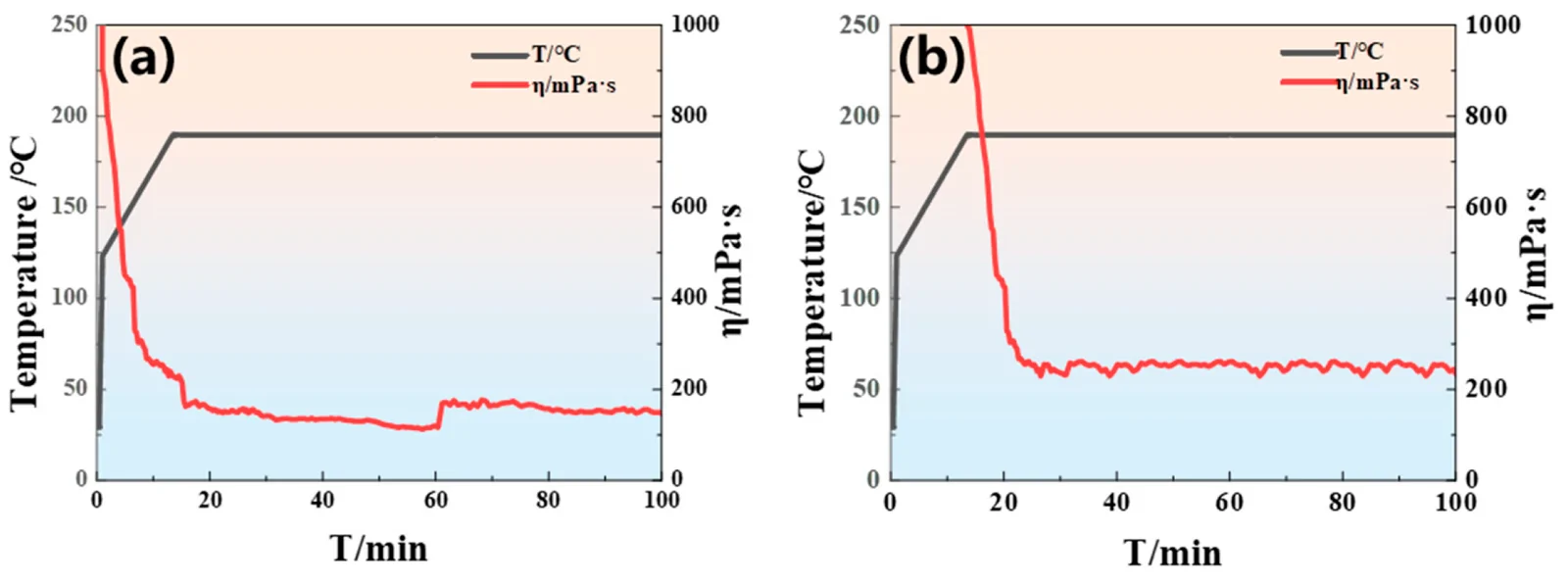
High-temperature rheology of 0.2 wt.% LX641 gels at volume ratios of (**a**) 100:0.3 and (**b**) 100:0.5. The samples were heated to 190 °C within 25 min and sheared at 190 °C until 100 min at 170 s^−1^.

The most severe rheological program was conducted using 0.2 wt.% LX641. The temperature was increased from approximately 30 to 190 °C during the first 25 min and then maintained at 190 °C from 25 to 100 min under a constant shear rate of 170 s^−1^. As shown in Figure 5, the apparent viscosities recorded at the end of the 100 min test were 172.85 mPa·s for the gel prepared at a base-fluid/crosslinker-solution volume ratio of 100:0.3 and 246.84 mPa·s for the gel prepared at a volume ratio of 100:0.5. Thus, the gels underwent a total programmed test of 100 min, including 75 min of isothermal shearing at 190 °C. The higher terminal viscosity obtained at the 100:0.5 volume ratio indicates that increasing the crosslinker dosage enhanced the resistance of the low-polymer gel network to combined thermal and shear-induced degradation. Recent studies have similarly demonstrated that ligand-regulated zirconium crosslinking, delayed gelation, and multilevel polymer networks can improve viscosity retention under high-temperature conditions relevant to ultra-deep reservoirs [9,19,20,21,22]. The present results demonstrate substantial high-temperature shear stability under the specified laboratory program; however, they do not establish long-term chemical stability or transport performance under actual reservoir conditions.

### 2.5. Oscillatory and Steady-Shear Rheology

**Figure 6:**
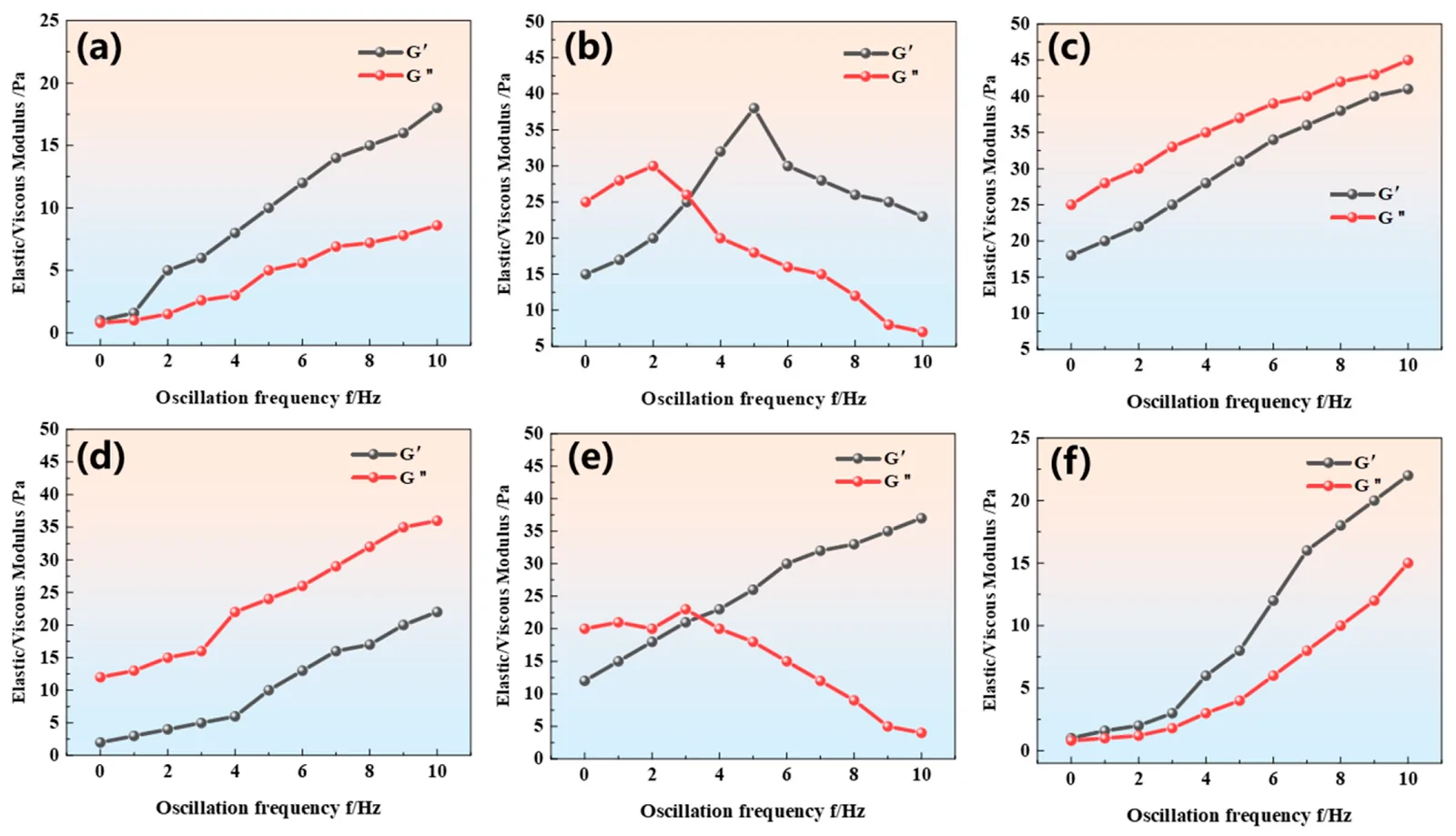
Frequency sweeps of LX641 gels at 25 °C and 0.1 Pa: (**a**–**c**) 0.3, 0.5, and 0.7 wt.% LX641 at a volume ratio of 100:0.5; (**d**–**f**) volume ratios of 100:0.3, 100:0.5, and 100:0.7 at 0.3 wt.% LX641.

As shown in Figure 6, frequency sweeps at 25 °C demonstrated that polymer concentration and crosslinker dosage altered the balance between elastic and viscous response. At 0.3 wt.% LX641 and a 100:0.5 volume ratio, G′ exceeded G″ over most of the measured frequency range, consistent with a gel-like response. Increasing polymer concentration increased the magnitude of the moduli, although the 0.7 wt.% sample displayed a more viscous-dominated response over part of the range. This non-monotonic behavior may reflect heterogeneous crosslinking, sample-preparation differences, or testing outside the optimal linear-viscoelastic window. Previous studies likewise show that network elasticity and microstructure, rather than viscosity alone, are important for evaluating proppant suspension [23,24]. Because amplitude-sweep data were not available, a unique mechanism cannot be assigned.

At fixed 0.3 wt.% LX641, the 100:0.5 ratio gave the clearest elastic-dominant response among the tested ratios. The 100:0.3 sample had lower moduli, whereas the 100:0.7 sample did not produce a proportional increase in elasticity. This supports an intermediate crosslinker dosage and is consistent with the practical observation that excessive metal crosslinkers can create locally dense, heterogeneous domains rather than a uniformly stronger network. These conclusions are comparative; future work should report amplitude sweeps, replicate frequency sweeps, and uncertainty intervals.

**Figure 7:**
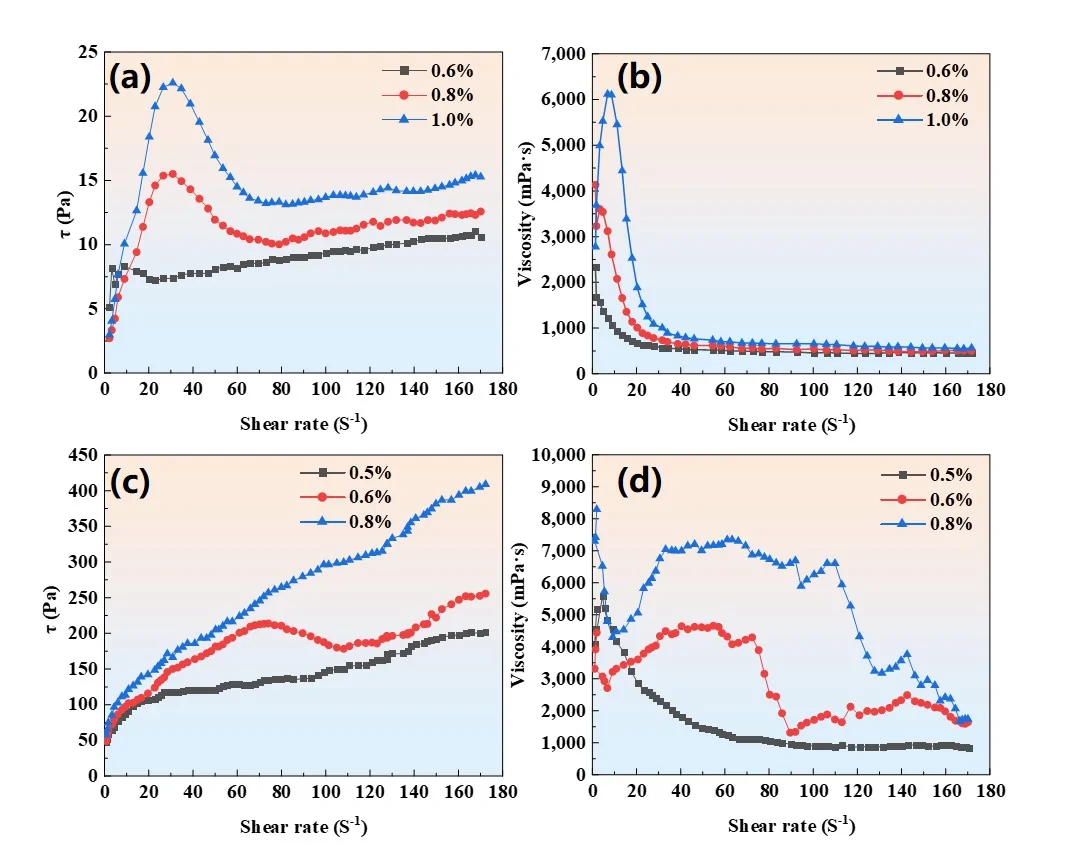
Steady-shear behavior of uncrosslinked LX641 solutions (**a**,**b**) and crosslinked gels (**c**,**d**): shear stress (**a**,**c**) and apparent viscosity (**b**,**d**).

As shown in Figure 7, both the uncrosslinked base fluids and crosslinked gels exhibited shear thinning in steady shear. In base fluids, increasing shear rate aligns polymer chains and decreases the contribution of physical entanglement to flow resistance. The crosslinked gels had much higher low-shear viscosity, followed by a pronounced viscosity decrease as the imposed shear rate increased, consistent with synthetic and hydrophobically associating polymer fracturing fluids [3,4,5,6,7]. The available flow curves were not fitted to a constitutive equation, and a quantitative yield stress was not determined. Accordingly, the data support pseudoplastic flow but not a specific Herschel–Bulkley or yield-stress parameter.

### 2.6. Static Fluid Loss, Gel Breaking, Proppant Suspension, and Core Damage

#### 2.6.1. Static Fluid Loss

As shown in Table 4, the static fluid-loss parameters increased with temperature from 60 to 100 °C. At 100 °C, the spurt loss was 2.098 × 10^−3^ m^3^ m^−2^, the fluid-loss rate was 0.0086 × 10^−3^ m min^−1^, and the fluid-loss coefficient was 0.0519 × 10^−3^ m min^−1/2^. Organic-metal, graphene-oxide, and nanosilica crosslinker studies have similarly shown that strengthening the gel network can improve filtration control or fluid retention [13,17,18]. Under the adopted calculation and unit conventions, all three parameters satisfied the corresponding requirements of SY/T 6376-2008, indicating effective static fluid-loss control under the specified laboratory conditions.

#### 2.6.2. Gel Breaking and Broken-Fluid Properties

As shown in Table 5, ammonium persulfate (APS) accelerated gel breaking at 70 °C. At 0.01 and 0.02 wt.% APS, the gel became thin after 1 h and visually clear after 2 h. At 0.03 and 0.04 wt.% APS, a clear fluid was observed after 1 h. No visible residue was observed in the broken fluids. For the fluid broken with 0.01 wt.% APS, the measured apparent viscosity, surface tension, and interfacial tension were 2.32 mPa·s, 22.022 mN m^−1^, and 1.36 mN m^−1^, respectively. Gel-breaking studies have shown that viscosity alone may not fully represent degradation or fracture-conductivity damage, supporting the need for complementary residual-solids and transport measurements [25]. These results indicate effective viscosity reduction and favorable interfacial properties, while the statement regarding residue is limited to visual observation rather than a gravimetric residual-solids measurement.

#### 2.6.3. Proppant Suspension

**Figure 8:**
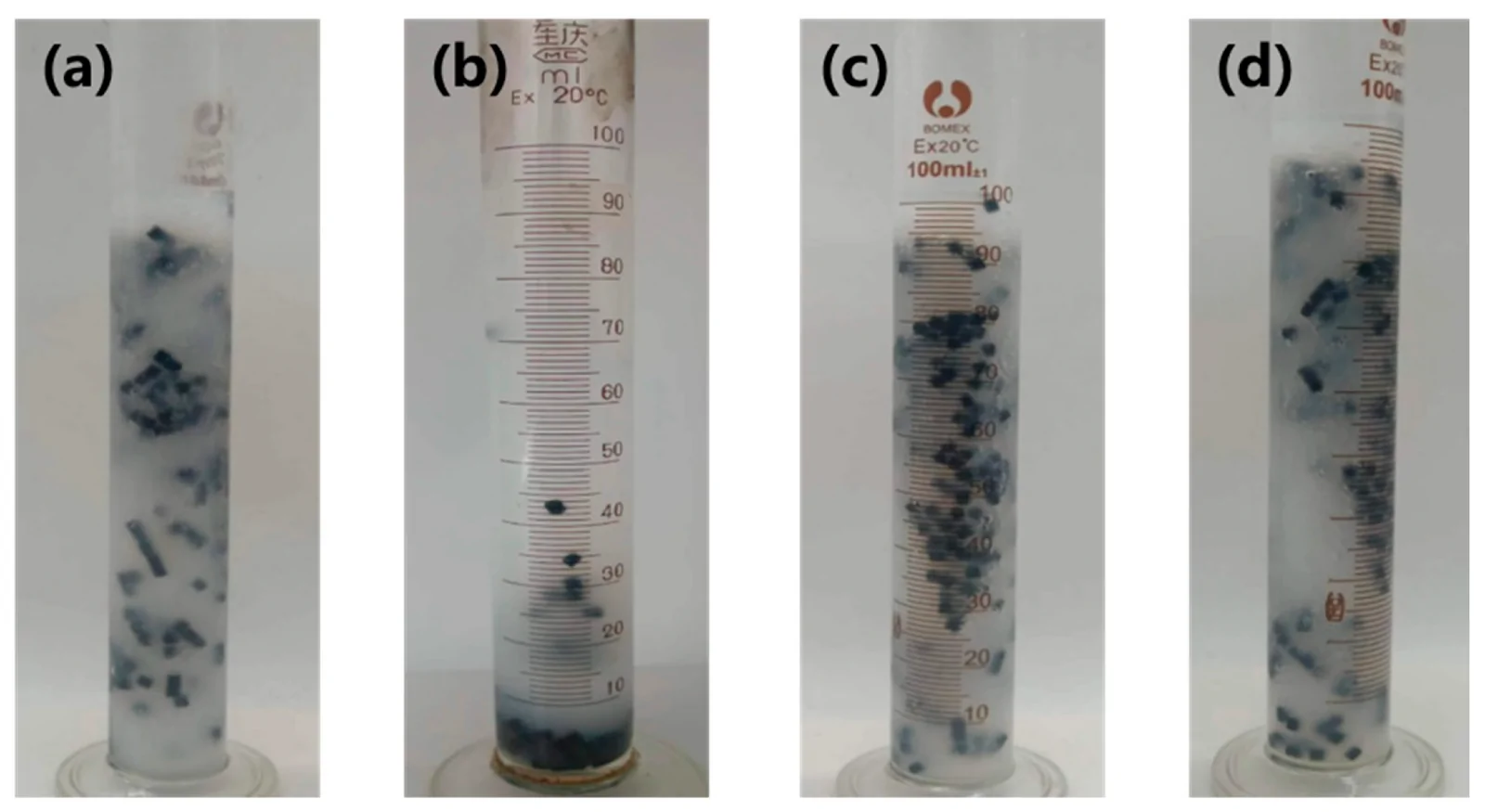
Static suspension of 10 wt.% 30-mesh ceramic proppant: uncrosslinked fluid initially (**a**) and after 12 h (**b**); crosslinked gel initially (**c**) and after 12 h (**d**).

As shown in Table 6, increasing the polymer concentration of uncrosslinked LX641 base fluids from 0.1 to 1.0 wt.% reduced the measured settling rate of 30-mesh ceramic proppant from 1.53 to 0.19 mm s^−1^. The 0.3 wt.% crosslinked gel had an apparent viscosity of 596.2 mPa·s and showed no visible settling during the observation.As shown in Figure 8, In the 12 h static photograph test, the proppant loading was 10 wt.%. Prior work indicates that elasticity and gel microstructure should be considered together with apparent viscosity when interpreting proppant suspension [23,24]. The result demonstrates static suspension under the stated laboratory condition but does not substitute for dynamic transport measurements in a fracture.

#### 2.6.4. Core-Permeability Damage

Two low-permeability natural cores were exposed to the filtered broken fluid prepared from the optimized gel. As shown in Table 7, permeability decreased from 25.69 to 21.65 mD for core D8-36 and from 12.96 to 11.06 mD for core Zuan 21-1, corresponding to damage rates of 11.98% and 13.65%. Permeability retention was therefore 88.02% and 86.35%, respectively. Fracturing-fluid damage can arise from residual polymers and solids, pore-throat retention, fluid sensitivity, and phase trapping [26,27]. These values indicate limited permeability impairment in the two tested plugs under the specified core-displacement protocol. However, the archived records do not contain the plugs’ field/formation source, depth, lithology, porosity, or dimensions, and only two cores were tested. The results therefore cannot be generalized to a specific reservoir or formation and should be interpreted as laboratory screening data. Formation-specific validation would require documented geological provenance, a larger statistically representative core set, mineralogical/petrophysical characterization, and comparison with a reference commercial fluid.

### 2.7. Gel Morphology and Proposed Coordination Mechanism

**Figure 9:**
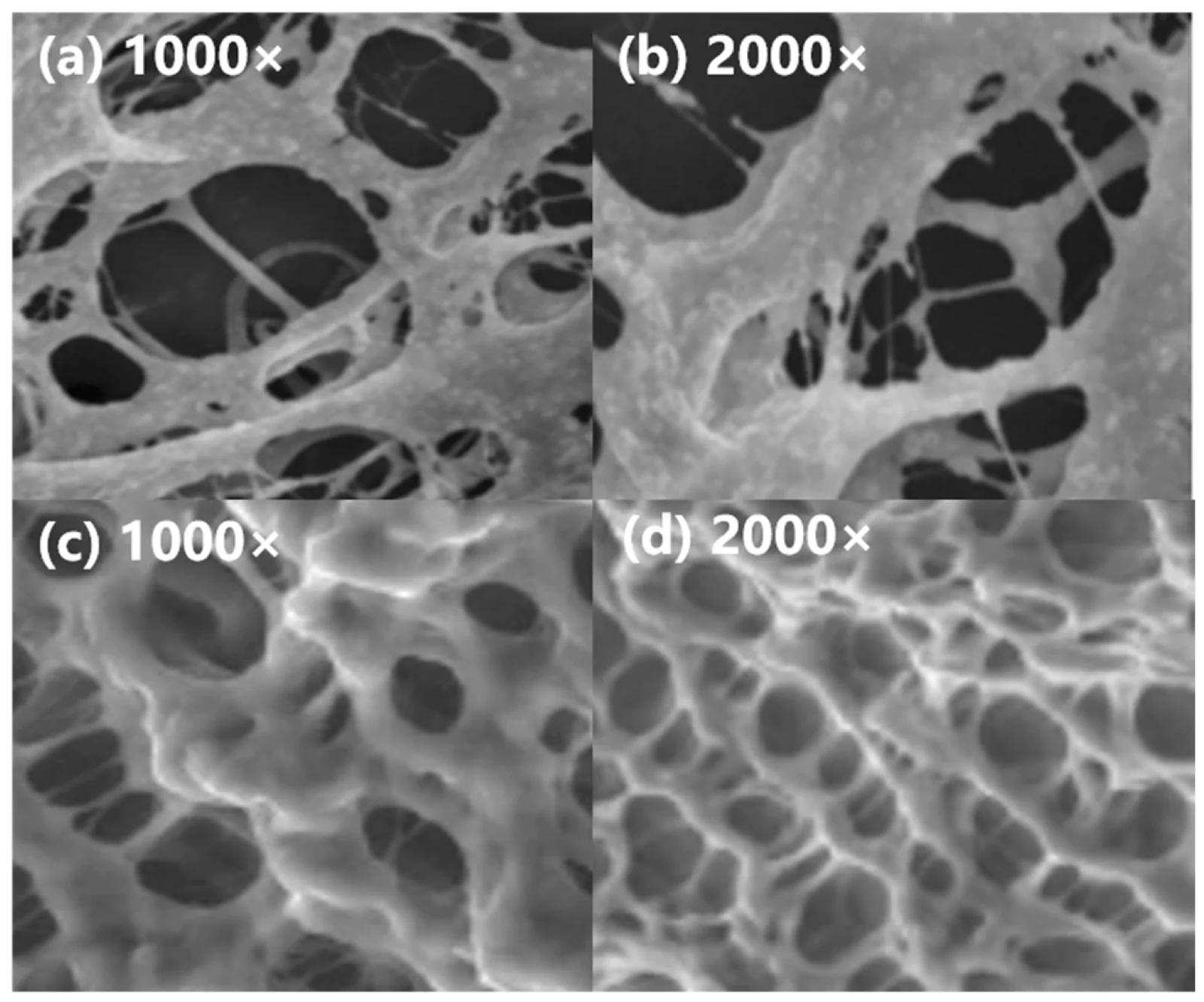
Representative electron-microscopy images of 0.3 wt.% LX641 before (**a**,**b**) and after (**c**,**d**) crosslinking at a base-fluid/crosslinker-solution volume ratio of 100:0.5.

Representative morphological differences between the uncrosslinked LX641 solution and the crosslinked fracturing gel are shown in Figure 9. Panels (a) and (b) show the 0.3 wt.% LX641 solution at nominal magnifications of 1000× and 2000×, respectively. The uncrosslinked polymer sample exhibited a relatively loose, irregular, and discontinuous morphology. Panels (c) and (d) show the corresponding crosslinked gel prepared at a base-fluid/crosslinker-solution volume ratio of 100:0.5 at nominal magnifications of 1000× and 2000×, respectively. Compared with the uncrosslinked sample, the crosslinked system exhibited a more interconnected and continuous porous morphology. This morphological contrast is consistent with the formation of a zirconium-coordinated polymer network. However, the observed structure may have been influenced by sample preparation and electron-microscopy conditions and should not be interpreted as a direct representation of the native hydrated gel network [16,17,18,28].

**Figure 10:**
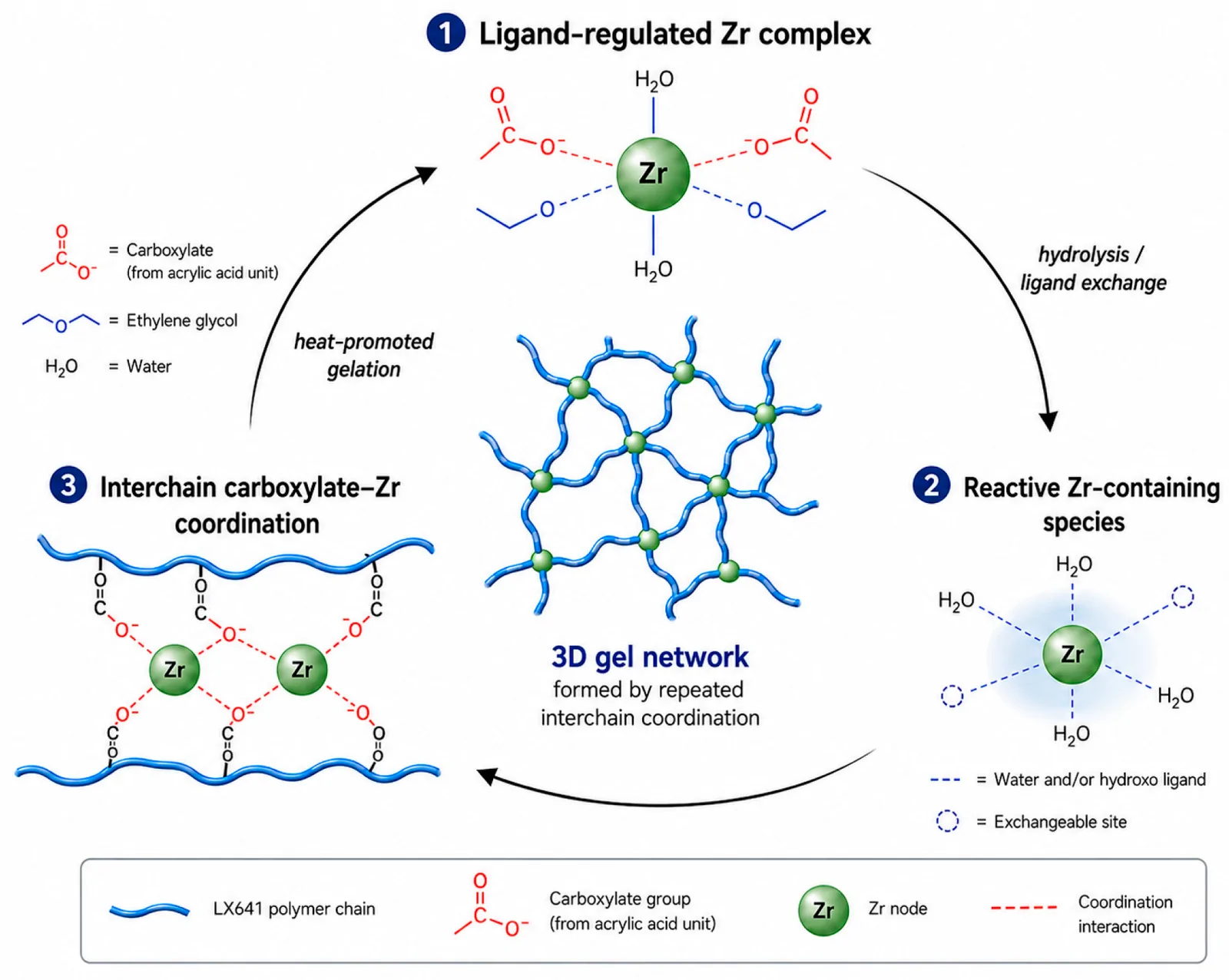
Proposed formation of a zirconium-coordinated LX641 gel network. The scheme is conceptual: zirconium-containing hydrolyzed complexes bridge carboxylate-bearing polymer chains, while the ligand environment regulates release and gelation kinetics.

LX641 contains acrylic acid units that provide carboxylate coordination sites, while partial hydrolysis of acrylamide units may supply additional carboxylates. AMPS contributes strongly hydrated sulfonate groups and steric protection, and DMDAAC introduces cationic functionality. In aqueous media, the organic zirconium complex undergoes ligand exchange and hydrolysis to generate reactive zirconium-containing species. As schematically illustrated in Figure 10, coordination between these species and carboxylate oxygen atoms on different polymer chains can connect the macromolecules into a three-dimensional gel network. Sodium lactate and ethylene glycol moderate zirconium reactivity during preparation and pumping, while elevated temperature accelerates the subsequent gelation process. This interpretation is consistent with studies of ligand-regulated zirconium crosslinkers, delayed multilevel networks, and broader reviews of gel fracturing fluids [9,10,11,12,13,14,19,20,21,22,29,30]. The mechanism is chemically plausible and consistent with FTIR and rheological behavior, but the exact zirconium nuclearity, ligand-exchange pathway, and distribution of crosslink sites remain to be directly determined.

### 2.8. Experimental Sensitivity and Literature Benchmarking

A response-span analysis was used to summarize the experimental sensitivity of crosslinker performance to the five single-factor synthesis variables. This analysis is descriptive rather than a formal global sensitivity analysis: each variable was changed individually while the remaining synthesis conditions were held at the corresponding experimental settings. The normalized response span was calculated as (*V*_max_ − *V*_min_)/*V*_max_ × 100%, using the measured apparent viscosity within each series. As summarized in Table 8, all five synthesis variables produced substantial changes in gel viscosity, with the largest response spans observed for synthesis temperature and the two ligand ratios. The pH series also showed a pronounced optimum at pH 7; the non-gelling pH 1 sample was excluded from the numerical span because no viscosity value could be defined.

Sensitivity was also evident at the formulation and operating levels. At 190 °C, increasing the base-fluid/crosslinker-solution volume ratio from 100:0.3 to 100:0.5 increased the apparent viscosity at 100 min from 172.85 to 246.84 mPa·s, an increase of approximately 42.8%. In the 30–120 °C programmed tests, increasing LX641 concentration from 0.2 to 0.4 wt.% increased terminal apparent viscosity from approximately 130 to 320 mPa·s. Salt response was ion-dependent, with the strongest viscosity reduction in AlCl_3_ and the weakest in NaCl. These trends identify crosslinker dosage, polymer loading, synthesis conditions, and ionic environment as the principal experimentally observed sensitivities of the present formulation.

A literature benchmark is provided in Table 9 to clarify the practical position of the present system. The comparison is intentionally limited to representative high-temperature zirconium-crosslinked gels for which key rheological conditions are reported in the cited studies [9,20,21,22]. Differences in polymer chemistry, crosslinker dosage, heating program, salinity, and test duration prevent a strict head-to-head ranking. Nevertheless, the comparison shows that the present system combines a comparatively low polymer concentration (0.2 wt.%) with substantial viscosity retention during a severe 190 °C programmed shear test.

### 2.9. Experimental Uncertainty, Reproducibility, and Study Limitations

The experimental results should be interpreted within several limitations. Independent replicate measurements were not available for all tests; therefore, standard deviations and confidence intervals are not reported for datasets lacking replicate measurements. The reported values should thus be regarded as representative measurements obtained under the specified laboratory conditions. Experimental uncertainty may also arise from sample preparation, pH adjustment, temperature programming, rheometer operation, and visual assessment of gelation, residue, and proppant settling.

The single-factor optimization approach does not quantify interactions among synthesis variables. In addition, an independent amplitude sweep was not performed before the frequency-sweep measurements; therefore, the linear viscoelastic region was not separately verified, and the oscillatory results are interpreted comparatively. The archived steady-shear datasets were also insufficient for statistically defensible constitutive-model fitting; therefore, power-law or Herschel–Bulkley parameters were not derived from the plotted curves. The proppant test reflects static suspension rather than dynamic transport in a propagating fracture, while the core-damage evaluation was limited to two low-permeability cores. FTIR and electron-microscopy observations support the proposed coordination mechanism but do not determine the exact zirconium nuclearity or ligand-exchange pathway.

These results are therefore limited to the tested laboratory formulations and operating conditions. Further work should include replicate measurements, multivariable optimization, dynamic proppant-transport testing, broader core evaluation, direct zirconium-speciation analysis, and comparison with commercial high-temperature fracturing fluids. The results are still to be confirmed by field application.

## 3. Conclusions

(1) A sodium-lactate/ethylene-glycol dual-ligand organic zirconium crosslinker was selected by ligand screening and single-factor optimization. The selected synthesis conditions were a zirconium oxychloride octahydrate/sodium lactate/ethylene glycol/water/NaOH mass ratio of 10:6:4:15:1.2, 55 °C, pH 7, and 4 h. FTIR supported oxygen-ligand coordination to zirconium, while microscopy of dried samples showed an aggregated porous morphology without establishing a unique solution-phase zirconium structure.
(2) The crosslinker formed coherent gels with LX641 at low polymer loading. In the 100 min high-temperature program, a 0.2 wt.% LX641 gel at a base-fluid/crosslinker-solution volume ratio of 100:0.5 retained 246.84 mPa·s after heating to 190 °C within 25 min and subsequent shearing at 190 °C for 75 min at 170 s^−1^. A separate 0.2 wt.% formulation retained approximately 130 mPa·s at the end of the 100 min 30–120 °C test.
(3) Under the laboratory conditions evaluated, the optimized gel maintained substantial viscosity in 0–4 wt.% inorganic salt solutions, showed low static fluid loss, suspended 10 wt.% 30-mesh ceramic proppant without visible settling for 12 h, and produced permeability-damage values of 11.98–13.65% in two natural core plugs. At 70 °C, 0.01 wt.% APS produced a visually clear, low-viscosity broken fluid within 2 h.
(4) The principal contribution is the demonstration that dual-ligand regulation of an organic zirconium crosslinker can support substantial high-temperature rheological performance at only 0.2 wt.% LX641. The conclusions remain laboratory-scale: statistical replication was incomplete, the linear viscoelastic region was not independently verified, the core study was not formation-specific, and dynamic transport and field trials were not conducted. Accordingly, field applicability requires further validation.

## 4. Materials and Methods

### 4.1. Materials

Zirconium oxychloride octahydrate (ZrOCl_2_·8H_2_O), ethylene glycol, sodium lactate, sodium hydroxide, hydrochloric acid, sodium chloride, calcium chloride, aluminum chloride, and ammonium persulfate were analytical-grade reagents and were used as received. These reagents were purchased from Tianjin Tianli Chemical Co., Ltd. (Tianjin, China). Industrial-grade LX641, an AM/AMPS/DMDAAC/AA tetrapolymer, together with flowback aid CHS-1, clay stabilizer COP-1, regulator TJ-1, and bactericide CJS-1, was supplied by the Changqing Exploration and Development Research Institute (Xi'an, China). Deionized water was used throughout.

### 4.2. Preparation of the Dual-Ligand Organic Zirconium Crosslinker

Deionized water and ZrOCl_2_·8H_2_O were added to a three-neck flask fitted with a reflux condenser and stirred until dissolved. The mixture was heated to 55 °C, after which sodium lactate and ethylene glycol were added slowly in sequence. The pH was adjusted to 7.0 with dilute NaOH, and the mixture was stirred at 55 °C for 4 h. The selected mass ratio of ZrOCl_2_·8H_2_O /sodium lactate/ethylene glycol/water/NaOH was 10:6:4:15:1.2.

### 4.3. Ligand Screening and Single-Factor Optimization

Organic zirconium formulations containing candidate hydroxy, carboxylate, and amine ligands were prepared and screened using crosslinker clarity, phase separation, gelation behavior, hanging-gel formation, and compatibility with LX641. Representative sodium lactate, ethylene glycol, glycerol, and mixed-ligand systems were subsequently compared quantitatively. For crosslinking tests, 100 mL of 0.5 wt.% LX641 base fluid was mixed with the crosslinker at the specified volume ratio and stirred at 500 rpm for 1 min. Gelation time and apparent viscosity were then recorded. After selection of the sodium lactate/ethylene glycol ligand combination, ligand ratios, synthesis time, temperature, and pH were varied individually, and gel apparent viscosity was used as the primary response variable for single-factor optimization.

### 4.4. Preparation of the LX641 Base Fluid and Crosslinked Gel

LX641 was dispersed in deionized water together with 0.5 wt.% CHS-1, 1.0 wt.% COP-1, 0.3 wt.% TJ-1, and 0.3 wt.% CJS-1. The viscosity of LX641 approached a plateau within approximately 20 min of mixing. For standardized laboratory testing, the fluids were sealed and aged for 24 h before use. The base-fluid pH was adjusted to 8.0 with HCl, and the organic zirconium crosslinker was added under high-speed mixing. Crosslinking ratios are expressed as the volume ratio of base fluid to crosslinker solution; thus, 100:x denotes 100 volume units of base fluid per x volume units of crosslinker solution.

### 4.5. FTIR and Microscopy

The crosslinker was vacuum-dried, ground, mixed with KBr, and analyzed by FTIR over 500–4500 cm^−1^ using a Nicolet spectrometer (Thermo Fisher Scientific, Waltham, MA, USA). The dried crosslinker and LX641 samples before and after crosslinking were imaged using a Quanta 3500 scanning electron microscope (FEI Company, Hillsboro, OR, USA). No NMR analysis was included in this study.

### 4.6. Rheological Measurements

Rheological measurements were performed using HAAKE MARS III, HAAKE R-600, and HAAKE RS-600 rheometers, as appropriate for the respective tests. Unless otherwise specified, high-temperature shear measurements were conducted at a constant shear rate of 170 s^−1^.

For the uncrosslinked LX641 solutions, the temperature was increased from 30 to 90 °C over 60 min. For the crosslinker-dosage comparison, gels containing 0.6 wt.% LX641 were prepared at base-fluid/crosslinker-solution volume ratios of 100:0.2, 100:0.3, and 100:0.4. The temperature was increased from 100 to 120 °C and was subsequently maintained at 120 °C. For the low-polymer tests, gels containing 0.2, 0.3, and 0.4 wt.% LX641 were prepared at a fixed base-fluid/crosslinker-solution volume ratio of 100:0.3. The temperature was increased from 30 to 120 °C during a 100 min programmed test under a constant shear rate of 170 s^−1^. The terminal apparent viscosity was recorded at the end of the 100 min test. For the 190 °C tests, gels containing 0.2 wt.% LX641 were prepared at base-fluid/crosslinker-solution volume ratios of 100:0.3 and 100:0.5. The temperature was increased from approximately 30 to 190 °C during the first 25 min and maintained at 190 °C from 25 to 100 min under a constant shear rate of 170 s^−1^. The terminal apparent viscosity was recorded at the end of the 100 min test. Oscillatory frequency sweeps were conducted at 25 °C over a frequency range of 0.1–10 Hz at an applied shear stress of 0.1 Pa. The storage modulus (G′) and loss modulus (G″) were recorded as functions of frequency. Because independent amplitude-sweep measurements were not available, the linear viscoelastic region was not separately verified, and the frequency-sweep results were therefore interpreted comparatively.

Steady-shear measurements were conducted at room temperature. Shear stress and apparent viscosity were recorded as functions of shear rate for the uncrosslinked LX641 solutions and the organic-zirconium-crosslinked gels.

### 4.7. Salt, Static Fluid-Loss, Gel-Breaking, and Proppant Tests

NaCl, CaCl_2_, and AlCl_3_ were added to the 0.3 wt.% LX641 gel at concentrations of 0–4 wt.% for the salt-response comparison. Static fluid-loss tests were carried out at 60, 80, and 100 °C under a differential pressure of 3.5 MPa using a high-temperature high-pressure filter press, following the adopted SY/T 6376-2008 procedure and calculation conventions.

For gel breaking, APS was added at 0.01–0.04 wt.% and the samples were held at 70 °C. Visual state and the presence or absence of visible residue were recorded after 0.5, 1, and 2 h. Apparent viscosity, surface tension, and interfacial tension were measured for the broken fluid prepared with 0.01 wt.% APS. Static proppant suspension was evaluated using 10 wt.% 30-mesh ceramic proppant over 12 h. Dynamic fracture-transport testing was not performed.

### 4.8. Core-Damage Test

Natural low-permeability core plugs D8-36 and Zuan 21-1 were used for laboratory permeability-damage screening. The plugs were saturated for 24 h with standard brine containing 2.0 wt.% KCl, 5.5 wt.% NaCl, 0.35 wt.% MgCl_2_, and 0.55 wt.% CaCl_2_. The optimized gel was broken with 0.01 wt.% APS at 70 °C, filtered, and approximately 300 mL of the filtered broken fluid was placed in the reservoir of a laboratory core-displacement apparatus. Initial brine permeability (k_0_) was measured, followed by reverse injection of 10 pore volumes of filtered broken fluid and subsequent forward brine flooding to determine permeability after treatment (k_1_). Permeability damage was calculated as η = (k_0_ − k_1_)/k_0_ × 100%. Detailed field/formation provenance, stratigraphic depth, lithology, porosity, plug dimensions, and the model designation of the displacement apparatus were not retained in the archived experimental records; therefore, the test is interpreted as a generic laboratory core-damage screening rather than a formation-specific evaluation.

## Figures and Tables

**Table 1:** Ligand screening of organic zirconium crosslinkers with LX641.

Ligand System	Best Volume Ratio (Base Fluid/Crosslinker Solution)	Gelation Time (min)	Viscosity (mPa·s)	Gel Observation
Sodium lactate	100:0.6	9	320.8	Hanging gel; brittle
Sodium lactate/ ethylene glycol	100:0.6	6	460.8	Coherent hanging gel; good elasticity
Sodium lactate/ glycerol	100:0.8	7	406.3	Hanging gel; good elasticity
Ethylene glycol/ glycerol	100:0.6	10	320.8	Hanging gel; brittle
Sodium lactate/ethylene glycol/glycerol	100:0.6	8	409.8	Hanging gel; brittle

**Table 2:** Single-factor optimization of the dual-ligand organic zirconium crosslinker.

Variable	Levels Examined	Selected Level	Response at Selected Level
ZrOCl_2_·8H_2_O/sodium lactate mass ratio	5:1–5:5	5:3	521.9 mPa·s
ZrOCl_2_·8H_2_O/ethylene glycol mass ratio	5:1–5:5	5:2	582.6 mPa·s
Reaction time	2–6 h	4 h	628.8 mPa·s
Synthesis temperature	45–85 °C	55 °C	689.6 mPa·s
Synthesis pH	2–9	7	828.8 mPa·s

**Table 3:** Effect of temperature on the gelation time of 0.3 wt.% LX641 gel at pH 8 and a volume ratio of 100:0.5.

Temperature (°C)	Gelation Time (min)
30	2.9
40	2.3
50	1.9
60	1.6
70	1.2
80	0.8

**Table 4:** Static fluid-loss parameters of the 0.3 wt.% LX641 gel.

Temperature (°C)	Fluid-Loss Rate (m min^−1^)	Spurt Loss (m^3^ m^−2^)	Fluid-Loss Coefficient (m min^−1/2^)
60	0.0018 × 10^−3^	0.006 × 10^−3^	0.0132 × 10^−3^
80	0.0059 × 10^−3^	0.519 × 10^−3^	0.0395 × 10^−3^
100	0.0086 × 10^−3^	2.098 × 10^−3^	0.0519 × 10^−3^

**Table 5:** Visual gel-breaking observations at 70 °C.

LX641 (wt.%)	APS (wt.%)	Base-Fluid/Crosslinker- Solution Volume Ratio	0.5 h	1 h	2 h
0.3	0.01	100:0.5	Gel	Thin gel	Visually clear; no visible residue
0.3	0.02	100:0.5	Gel	Thin gel	Visually clear; no visible residue
0.3	0.03	100:0.5	Thin gel	Visually clear; no visible residue	-
0.3	0.04	100:0.5	Thin gel	Visually clear; no visible residue	-

**Table 6:** Apparent viscosity and static proppant-settling behavior.

Fluid	LX641 (wt.%)	Apparent Viscosity (mPa·s)	Settling Rate (mm s^−1^)
Base fluid	0.1	16.2	1.53
Base fluid	0.3	56.8	0.89
Base fluid	0.5	72.8	0.33
Base fluid	0.8	92.1	0.26
Base fluid	1.0	108.5	0.19
Crosslinked gel	0.3	596.2	No visible settling

**Table 7:** Core permeability before and after treatment with the broken fluid.

Core	Initial Permeability (mD)	Permeability After Treatment (mD)	Damage Rate (%)	Permeability Retention (%)
D8-36	25.69	21.65	11.98	88.02
Zuan 21-1	12.96	11.06	13.65	86.35

**Table 8:** Descriptive sensitivity of gel apparent viscosity to single-factor crosslinker synthesis variables.

Variable	Tested Range	Selected Level	Measured Viscosity Range (mPa·s)	Normalized Response Span (%)
ZrOCl_2_·8H_2_O/sodium lactate mass ratio	5:1–5:5	5:3	156.2–521.9	70.1
ZrOCl_2_·8H_2_O/ethylene glycol mass ratio	5:1–5:5	5:2	156.8–582.6	73.1
Reaction time	2–6 h	4 h	267.5–628.8	57.5
Synthesis temperature	45–85 °C	55 °C	167.5–689.6	75.7
Synthesis pH	2–9	7	325.6–828.8	60.7

**Table 9:** Literature benchmark of representative high-temperature zirconium-crosslinked fracturing-gel systems.

System	Polymer Loading	Crosslinker	High-Temperature Shear Conditions	Retained Viscosity (mPa·s)	Damage Data	Ref.
Present study	LX641, 0.2 wt.%	Dual-ligand organic Zr	190 °C; 170 s^−1^; 100 min	246.84	11.98–13.65% (2 cores)	This work
Xin et al.	HPAM, 0.6 wt.%	Organic Zr	200 °C; 100 s^−1^; 90 min after heating	61.43	NR	[9]
Zhao et al.	Weighted gel; thickener 0.8 wt.%	Delayed-crosslinking gel system	200 °C; 100 s^−1^; duration NR	Meets reported temperature/ shear requirement	NR	[20]
Xu et al.	Supramolecular enhanced polymer; concentration NR in abstract	Multisite organic Zr	200 °C; shear rate/time NR	71	NR	[21]
Huang et al.	Supramolecular polymer system, 0.4 wt.%	Zr crosslinker, 0.1 wt.%	200 °C; 170 s^−1^; 120 min	66	NR	[22]

## Data Availability

The data supporting the findings of this study are available from the corresponding author upon reasonable request.

## Nomenclature

**Symbol/Abbreviation**	**Definition**
AA	Acrylic acid
AM	Acrylamide
AMPS	2-Acrylamido-2-methylpropane sulfonic acid
APS	Ammonium persulfate
DMDAAC	Dimethyldiallylammonium chloride
G′	Storage modulus
G″	Loss modulus
HPAM	Partially hydrolyzed polyacrylamide
K_0_	Initial core permeability
K_1_	Permeability after treatment
PV	Pore volume
η	Permeability damage rate
$\overset{\cdot }{\gamma }$	Shear rate
